# Are some people at increased risk of paracetamol-induced liver injury? A critical review of the literature

**DOI:** 10.1007/s00228-017-2356-6

**Published:** 2017-10-24

**Authors:** Thomas M. Caparrotta, Daniel J. Antoine, James W. Dear

**Affiliations:** 1 Speciality Registrar Clinical Pharmacology and Therapeutics, NHS Lothian, Edinburgh, UK; 2 MRC Centre for Inflammation Research, University of Edinburgh, Edinburgh, UK; 3 University/BHF Centre for Cardiovascular Science, University of Edinburgh, Edinburgh, UK

**Keywords:** Paracetamol, Acetaminophen, Hepatotoxicity, Genomics, DILI

## Abstract

**Purpose:**

Paracetamol is one of the world’s most commonly used drugs. In overdose, it is well established to be hepatotoxic. The aim of this review was to identify factors that have been, or actually are, associated with the development of liver injury after paracetamol exposure in humans.

**Method:**

Google Scholar and PubMed were searched on various dates between December 2016 and March 2017. Papers identified had their references analysed for further studies that might be relevant.

**Results:**

At the time of writing, there was little good quality clinical evidence—from studies of paracetamol overdose or therapeutic use—to suggest that any groups of people are relatively protected from, or are at greater risk of, liver injury. The factors that were historically used to indicate higher risk in the UK have no good quality clinical evidence to support their re-introduction into clinical practice. The safe (and still effective) oral dose of paracetamol in patients weighing less than 50 kg has not been established.

**Conclusion:**

There is no patient group that is unequivocally at elevated risk of paracetamol-induced liver toxicity. We propose two clinical scenarios that warrant further research. Firstly, there is a need to establish whether the dose of paracetamol should be reduced in patients with low body weight. Secondly, if or when genomic information regarding individual patients becomes readily available to inform prescribing, we propose the contribution of the genome to paracetamol toxicity should be re-investigated with robustly designed studies. Such studies could enhance the safe use of one of the most frequently taken drugs.

**Electronic supplementary material:**

The online version of this article (10.1007/s00228-017-2356-6) contains supplementary material, which is available to authorized users.

## Introduction

### Paracetamol

Paracetamol (acetaminophen, APAP) is an analgesic for mild to moderate pain and an anti-pyretic [1]. It is widely available for purchase without prescription and in recommended dosages is believed to have an excellent safety profile [1]. However, in overdose, paracetamol is well known to be hepatotoxic [2]. Fortunately, the toxicity of paracetamol overdose is effectively attenuated by administration of the antidote acetylcysteine (NAC), at least if administered within 8 h of the overdose [3].

In this review, we will discuss factors that have been, or actually are, associated with the development of liver injury following paracetamol exposure. Our objective is to critically identify genotypes and phenotypes that put patients at higher or lower risk of toxicity. In this review, we define acute liver injury as having serum alanine aminotransferase activity (ALT) of 100–1000 IU/L and hepatotoxicity as ALT > 1000 IU/L.

### Epidemiology of overdose

Paracetamol poisoning is an important clinical entity as it accounts for 50% of poisonings in the UK and 10% in the USA [4, 5]. In the UK alone, it results in 82,000–90,000 hospital presentations per year [2]. Toxicity generally falls into two categories: intentional overdose and unintentional overdose (therapeutic misadventure). There are reports of rare cases of toxicity following dosing within the therapeutic range, although there is debate about whether this latter category truly exists. Indeed a review article concluded that in prospective trials, therapeutic doses of paracetamol do not cause toxicity, and notes that only in retrospective studies does therapeutic toxicity occur and this is likely due to the biases inherent in retrospective analysis [2, 6, 7]. Around 150–250 deaths occur directly due to paracetamol poisoning each year in the UK and these generally occur in patients who presented late to hospital, took a massive overdose, those who staggered their overdose, or who took an unintentional overdose [8, 9]. Since 2012, new guidelines on the treatment of paracetamol poisoning have been implemented in the UK [10]. The revised guidelines recommend treating all patients with a paracetamol concentration above a single treatment line on the paracetamol nomogram (the ‘100 milligram (mg) per litre (L) at four hours after overdose treatment line’) and treating all patients with a staggered overdose or uncertain time of ingestion [10]. This has been reported to have resulted in a 7% absolute increase in the number of patients admitted to hospital for paracetamol poisoning [2].

### Paracetamol metabolism and toxicity

**Fig. 1:**
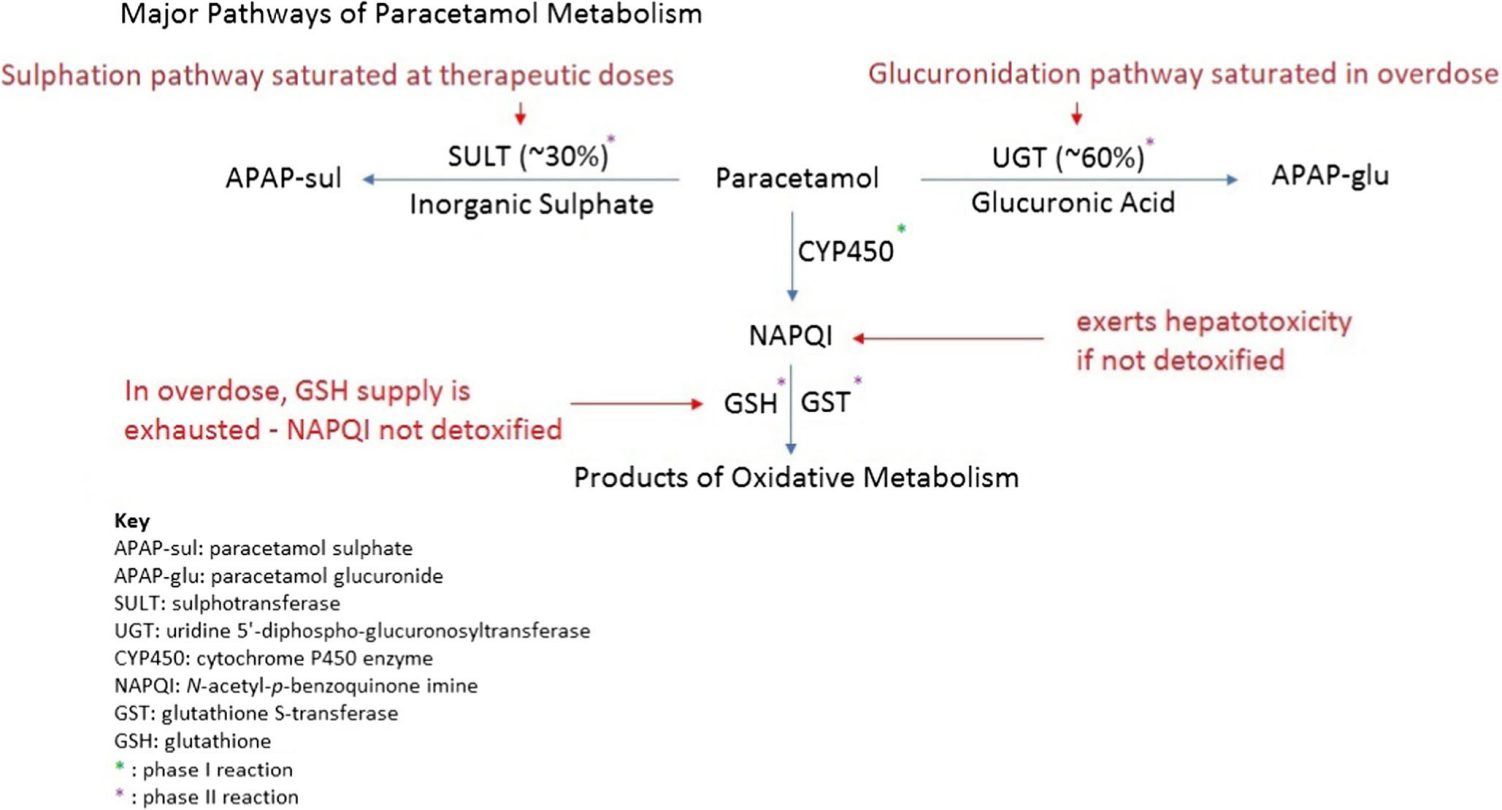
Major pathways of paracetamol metabolism

Following oral administration absorption is rapid, with a paracetamol peak plasma concentration (*C*_max_) occurring at ~ 1 h post-administration. However, absorption may be prolonged in a patient taking an overdose [11, 12]. The mean systemic bioavailability of paracetamol following oral administration is ~ 75% [13, 14]. Following delivery of a therapeutic dose of paracetamol to a healthy person, the majority of paracetamol is directly conjugated in the liver by phase II enzymes to form glucuronide (APAP-glu) and sulphate (APAP-sul) derivatives. Early pharmacokinetic studies demonstrated that following a therapeutic paracetamol dose, 55% is excreted as the glucuronide, 30% as the sulphate and 4% is excreted as the products of oxidative metabolism. The metabolic half-life (t_1/2_) is 1.5–2.5 h but may be prolonged following paracetamol overdose [13]. The sulphation pathway is saturable at therapeutic doses whereas the glucuronidation pathway only becomes saturated in overdoses (see Fig. 1 for a summary of the major pathways of paracetamol metabolism) [15, 16].

A smaller proportion is oxidised by phase I reactions catalysed by mixed function oxidases (cytochrome P450, CYP450), nicotinamide adenine dinucleotide phosphate oxidase and oxygen [13, 17]. These phase I oxidative reactions metabolise 2–10% of paracetamol to a reactive intermediate, *N*-acetyl-*p*-benzoquinone imine (NAPQI) which mediates the hepatotoxic effect of paracetamol. The proportion of the dose metabolised by this pathway increases in overdose and, potentially, in the presence of other pathological/physiological states [13, 18–20]. In humans, the CYP450 enzyme that predominately catalyses the formation of NAPQI is CYP2E1, although in vitro CYP3A4, CYP2D6 and CYP1A2 have also been implicated in the formation of the toxic metabolite [18–20]. NAPQI is a strong oxidising agent and has the potential to cause liver and kidney damage by reacting with cellular and mitochondrial proteins [21]. However, following therapeutic doses, a healthy liver is able to detoxify NAPQI rapidly due to the presence of reduced glutathione (GSH). The sulfhydryl group of GSH binds NAPQI, which is subsequently excreted in the urine as mercapturic acid or cysteine conjugates [13, 20]. This leads to depletion of hepatocyte GSH [13]. In paracetamol overdose, a greater proportion of the dose is shunted to the oxidative pathway which results in greater NAPQI formation [13]. When large quantities of NAPQI are formed, hepatocyte glutathione can be critically depleted, meaning that excess NAPQI is not detoxified and cell injury occurs [13]. NAPQI formation can be monitored indirectly through the presence of circulating paracetamol-protein adducts (PPA). There is evidence to suggest that PPA are formed in healthy individuals at therapeutic doses, particularly following repeated doses, suggesting that not all NAPQI is scavenged by glutathione even during therapeutic administration (see Fig. 1 for a summary of the major pathways of paracetamol metabolism) [22, 23].

It is widely assumed that paracetamol doses in the therapeutic range are not toxic. However, a 145-patient randomised controlled trial (RCT) reported that 4 g of paracetamol daily in divided doses for 4 days was associated with an increase in ALT activity in a significant proportion of patients when compared to placebo, although the clinical significance of this elevation in ALT is unclear [24]. The patients in this trial were admitted to a trials unit with a possible change in their sleep patterns and diet that could have caused fluctuations in ALT. However, another 205-patient RCT reported that, although a minority of patients did experience an ALT rise with 4 g paracetamol daily, this rise resolved following further days of dosing [25]. Therefore, there is conflicting evidence regarding the effect of therapeutic doses of paracetamol on liver enzymes and the significance of an increase in ALT activity.

A retrospective analysis of 9479 referrals to multi-national liver transplant units gave an event rate of 3.3 million treatment years for *non-overdose* paracetamol as the aetiology for acute liver failure requiring transplant. However, cases were assigned as ‘non-overdose’ by an expert committee if there was no history of overdose and a non-toxic plasma paracetamol concentration (PPC), meaning that some of these cases may in fact have been concealed overdoses if a history of overdose was not given and enough time had elapsed for PPC to become undetectable [26].

In summary, the risk of clinically significant liver injury at therapeutic doses of paracetamol is very low. Despite this, in this review, we consider evidence from both therapeutic dosing and overdose studies because pre-clinical data suggest everyone would develop liver injury if the dose of paracetamol was sufficiently high for that individual. Therefore, a key question is whether certain individuals have an ‘inflection point’ for clinically important paracetamol toxicity at a dose within or close to the recommended therapeutic dose. The dose-response relationship of paracetamol and liver injury is in contrast to so-called idiosyncratic drug-induced liver injury, which is restricted to a subset of susceptible individuals.

## Method

### Literature search strategy

Google Scholar and PubMed were searched on various dates between December 2016 and March 2017, ending on 15th March 2017 using the following terms, and variants thereof, and all articles discovered were considered for inclusion: (paracetamol OR acetaminophen hepatotoxicity) AND genetic differences/polymorphisms OR children/neonates/young people/paediatric OR older people/elderly/geriatric OR nutritional state/malnutrition OR body weight/low body weight OR alcohol/alcohol-dependence OR drug interactions/potentiation OR liver disease/chronic liver disease/liver failure.

Furthermore, papers identified had their references analysed for any further studies that might be relevant to this review.

### Inclusion criteria

1. Studies with human participants and studies involving actual human tissue samples where liver injury or hepatotoxicity is detailed, including mechanistic studies, case series, observational studies and randomised controlled trials, reviews, and systematic review articles

### Exclusion criteria

1. Studies involving animals
2. Studies not published in the English language
3. Studies where it was not possible to ascertain from the title or abstract that the inclusion criteria were met
4. Studies already included in other published reviews where the review is cited in the text

## Results—are some people at higher risk of paracetamol toxicity?

### Genetic differences

A number of enzymes are involved in the metabolism of paracetamol. Variations in some of these may contribute directly or indirectly to increase an individual’s risk of toxicity and may account for the wide inter-individual variability of paracetamol metabolism reported in some studies [17, 20, 27]. Although investigations have been undertaken looking at enzymes in isolation, little has been studied about how genetic differences act together to alter an individual’s risk of paracetamol toxicity [16].

### Uridine 5′-diphospho-glucuronosyltransferase and Gilbert’s syndrome

**Fig. 2:**
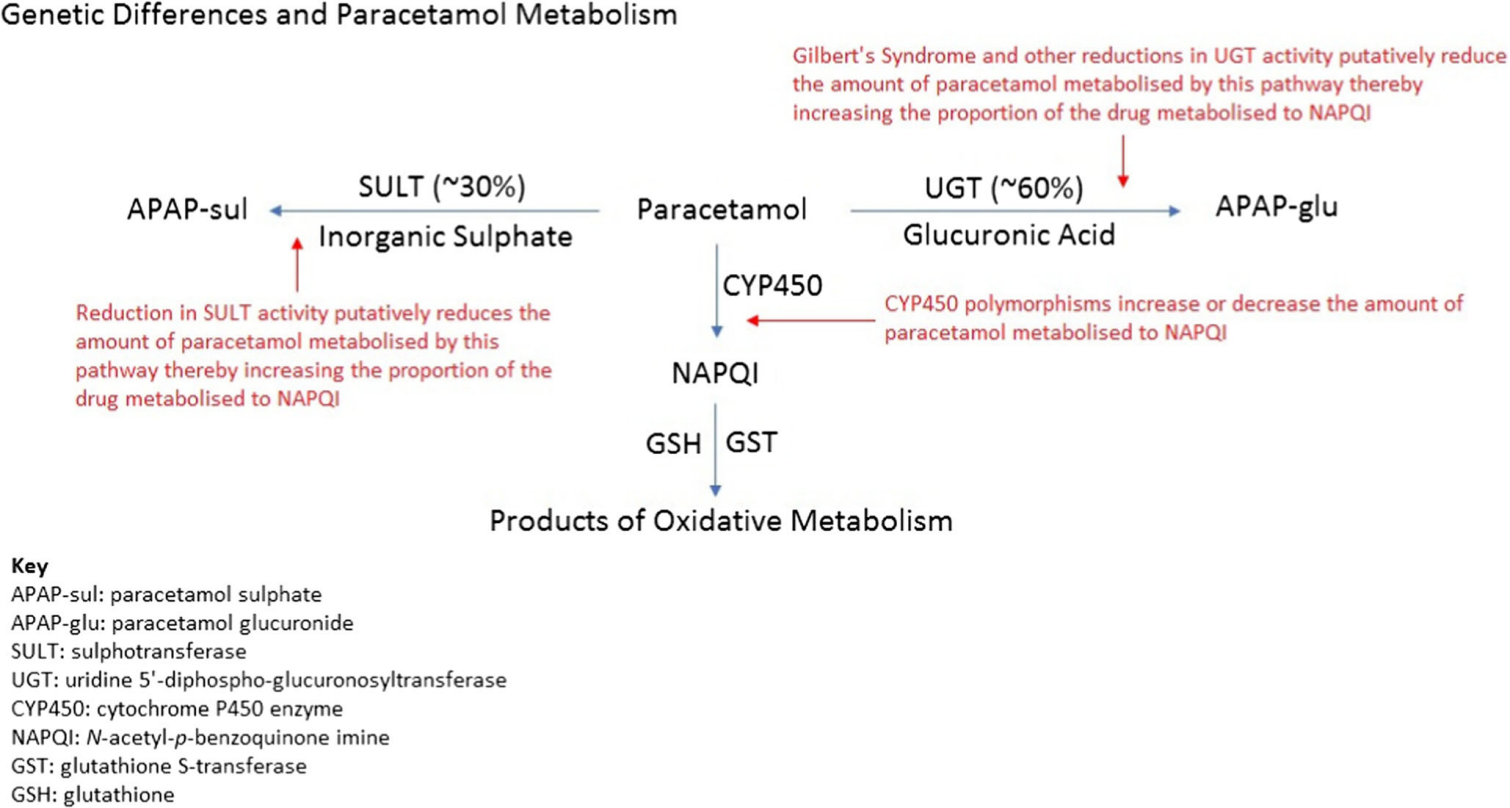
Genetic differences and paracetamol metabolism

Uridine 5′-diphospho-glucuronosyltransferase (UGT) catalyses the major metabolic pathway of paracetamol by glucuronidating its phenolic group to form APAP-glu, which is excreted renally [28, 29]. There are at least 15 isoforms of UGT in humans; UGT1A1, UGT1A9, UGT1A6 and UGTB15 are reported to glucuronidate paracetamol. Polymorphisms of these genes or associated regulatory sequences, or copy number variations (CNV) may contribute to a reduction in UGT activity or indeed loss of function and, therefore, reduce paracetamol glucuronidation. Theoretically, this could result in increased fractional oxidation and an increased propensity to toxicity (see Fig. 2 for the metabolic pathways affected by UGT) [17, 30, 31]. Conversely, the UGT1A rs8330 polymorphism has been associated with increased levels of paracetamol glucuronidation. A study in human liver tissue samples found that UGT1A rs8330 G polymorphism was consistently associated with higher rates of paracetamol glucuronidation. The same polymorphism was subsequently demonstrated to have a significantly lower incidence in patients who developed hepatotoxicity from unintentional paracetamol overdose when compared to liver failure from other causes [32]. This suggests that the higher rates of glucuronidation associated with this UGT1 polymorphism protect against paracetamol hepatotoxicity by decreasing the fractional proportion of paracetamol available for oxidation.

### Gilbert’s syndrome

There is heterogeneously reduced UGT activity in patients with Gilbert’s syndrome (present in 5–7% of the population, varies by ethnicity and by diagnostic method). The concern surrounding Gilbert’s syndrome and paracetamol is based on the putative theory that a reduction in the glucuronidation of paracetamol leaves a greater fraction of the drug available to be oxidised, potentially causing toxicity.

Although there are no case reports or case series suggesting increased risk of paracetamol-related toxicity in people with Gilbert’s syndrome, a number of controlled studies have investigated the effect of this reduction in UGT activity on paracetamol metabolism. There are conflicting results possibly due to differences in experimental design.

Three pharmacokinetic (PK) studies (total of 30 patients) have suggested that those with Gilbert’s syndrome have lower paracetamol glucuronidation [28, 33, 34]. One of these studies demonstrated a 1.7-fold higher recovery of toxic metabolites in those with Gilbert’s syndrome compared to controls [33], although a larger study only found a higher rate of recovery of toxic metabolites in those patients stratified by having the lowest UGT activity [28]. A different PK investigation, comparing Gilbert’s patients and controls, demonstrated no difference between ratios of metabolites [29]. Thus, the findings of these studies are inconsistent.

None of these investigations comprehensively assess the effect of Gilbert’s syndrome on the full PK and metabolite profiles of paracetamol. Furthermore, no studies exist investigating whether there is an over-representation of patients with Gilbert’s syndrome in those who develop toxicity following paracetamol overdose. There is no good quality clinical evidence to suggest that a diagnosis of Gilbert’s syndrome puts patients at risk of toxicity at therapeutic doses of paracetamol, and the assertion that patients are at greater risk of toxicity following overdose remains theoretical (see Fig. 2 for the pathways metabolic affected by UGT).

### Sulphotransferase superfamily

Sulphotransferase (SULT) enzymes catalyse the addition of a sulphate to the phenolic group of paracetamol to form APAP-sul (30–44% of total dose at therapeutic doses) [13, 17]. SULT1A1 is believed to be the major enzyme associated with paracetamol sulphation, although SULT1A3/4 and SULT1E1 have also been implicated in the foetus and neonates [20, 35, 36].

In theory, reduced paracetamol sulphation due to reduced sulphotransferase activity could lead to increased paracetamol oxidation to NAPQI. No PK studies investigating paracetamol metabolism in humans with SULT polymorphisms or CNVs have been conducted. Moreover, no studies have been undertaken in controlled circumstances to demonstrate whether a reduction in sulphotransferase activity causes increased paracetamol oxidation or increased propensity to paracetamol toxicity at either therapeutic or supra-therapeutic doses. There is no good quality clinical evidence to suggest that a reduction in sulphotransferase activity affects paracetamol metabolism or increases the risk of paracetamol toxicity (see Table 1 in the supplementary data for a summary of the evidence and see Fig. 2 for the metabolic pathways affected by SULT).

### Cytochrome P450

CYP2E1 is likely to be the most important enzyme involved in the bioactivation of paracetamol to NAPQI [20]. The coding portion of the gene is well conserved across species, although polymorphisms have been described in the non-coding portions of the gene [37]. There are significant inter-ethnic differences in the polymorphism frequency, but it is unclear whether these result in a clinically relevant phenotype (see Table 2 in the supplementary data for a summary of the evidence) [37]. It is widely accepted that alcohol use and a number of pharmacological agents, for example isoniazid, induce expression of CYP2E1 (see section “Potential drug interactions with paracetamol metabolism” and section “Alcohol use”).

CYP2D6 is a minor enzyme associated with the oxidation of paracetamol; however, it constitutes the most variable of the CYP450 genes as well as the most complex CYP450 locus [38]. As with CYP2E1, there are substantial inter-ethnic differences in CYP2D6 across populations, ranging from complete loss of function or reduction in functional activity (25–70% of the population, dependent on ethnic origin and geographical location) to extensive, ultra-rapid metabolisers (1.5–9.3% of the population) (see Table 3 in the supplementary data for a summary of the evidence) [17, 38].

DNA samples obtained from patients enrolled by the Acute Liver Failure Study Group suggest the rs776746 polymorphism in CYP3A5 is associated with increased bioactivation of paracetamol via increased enzyme activity. This polymorphism was retrospectively found to be over-represented in patients developing hepatotoxicity following intentional paracetamol overdose [39]. Although not traditionally believed to be a CYP450 enzyme involved in paracetamol oxidation, it may be that possession of this allele allows CYP3A5 to use paracetamol as a substrate and hence enhance its bioactivation with resultant liver injury.

No prospective studies have been conducted in humans in controlled circumstances to demonstrate whether CYP450 polymorphisms lead to an increase or a decrease in paracetamol oxidation nor have there been studies demonstrating that CYP450 polymorphisms in humans increase or decrease the risk of paracetamol toxicity before or after an overdose. There is no good quality clinical evidence to suggest that CYP450 polymorphisms influence paracetamol toxicity (see Fig. 2 for the metabolic pathways affected by CYP450).

### Glutathione s-transferase

When formed by oxidation of paracetamol, NAPQI is rapidly detoxified by conjugation with reduced GSH via reaction with GSH’s sulfhydryl group. This reaction occurs both spontaneously (non-enzymatically) and enzymatically, mediated by glutathione s-transferase (GST) [40]. GST is polymorphic and three polymorphisms decrease or abolish GST activity, although it is unclear whether possession of these polymorphisms leads to an increased propensity for paracetamol toxicity [41]. There is wide inter-ethnic variation in the frequency of GST polymorphisms [17]. There is no good quality clinical evidence from controlled studies in humans to suggest that GST polymorphisms affect paracetamol toxicity either at therapeutic or supra-therapeutic doses (see Table 4 in the supplementary data for a summary of the evidence).

### CD44

Two human genetic studies have suggested that polymorphisms in the CD44 gene may account for variations in toxicity following paracetamol overdose [39, 42]. CD44 is expressed on leukocytes and has a role in mediating the innate immune response. In one study, the CD44 rs1467558 polymorphism was reported to be significantly associated with an ALT rise in independent cohorts of patients given a therapeutic dose of 4 g/day of paracetamol over 7 days [42]. In silico modelling suggests that rs146558 is a nonsynonymous single nucleotide polymorphism that leads to disruption in protein function. Furthermore, the same polymorphism was found to be over-represented in patients developing acute liver injury following high doses of paracetamol taken unintentionally over several days [39]. Although CD44 is not involved in paracetamol pharmacokinetics, it is implicated in the inflammatory response to liver injury [43].

### Inter-individual variability and ethnicity

A number of studies have investigated the inter-individual variation of paracetamol metabolism secondary to ethnicity. Most of these studies are relatively small. The results of these studies are mixed, and due to study design, it is difficult to draw comparisons or firm conclusions (see Table 5 in the supplementary data for a summary of the evidence).

Although there may be differences in the metabolism of paracetamol between people of different ethnicity, it is unclear whether these are of any toxicological significance. There is no good-quality evidence to suggest that people of different ethnic backgrounds are of greater risk of toxicity at either therapeutic or supra-therapeutic doses.

### Age

#### Young age

A number of studies have investigated the difference in paracetamol metabolism between neonates, children and adults and whether this affects their propensity to paracetamol-induced toxicity.

Five PK studies all reported that children under 12 years of age metabolise paracetamol predominately by sulphation compared with adults (*n* = 114 children in total) and that the adult pattern of metabolism is present beyond the age of 12 [44–48]. These findings are supported by a critical review of the literature [49]. Three of the studies did not detect the products of oxidative metabolism following *therapeutic* dosing in children [44, 46, 48]; adults have measurable products of oxidative metabolism following therapeutic dosing. One study found that paracetamol t_1/2_ was prolonged in very early pre-terms (28–32 weeks’ gestational age), suggesting that the dose interval may need to be extended in this group, although only a single dose was given [47]. Another study reported that, in children with fever, the area under the serum concentration-time curve (AUC) increased by 13–44% from first dose to steady state implying that paracetamol may accumulate in this patient group although no ‘hepatotoxicity’ was demonstrated [48]. A critical review of the literature suggested that paracetamol clearance is lower in neonates but that neonates and children are capable of NAPQI formation, particularly following *multiple sequential overdoses*, and that toxicity can occur; they conclude that the differences in metabolism are due to immature glucuronidation and that sulphation is the major route of excretion [49].

Two case reports suggest that neonates and children have a reduced propensity to toxicity following paracetamol overdose due to differences in metabolism [50, 51]. One case report states that the recovery of the products of oxidative metabolism in a child was 12% following overdose compared to an expected rate of 35–43% reported in adults; although this patient developed hepatotoxicity and was treated with NAC and dialysis, it was suggested that this case was less clinically severe than would be expected in an adult [51]. Retrospective case series of paediatric patients referred to national poisons centres also report a lower rate of liver injury and hepatotoxicity in children compared with adults following *single acute overdose* [52–55]. However, further retrospective case series have identified liver injury and hepatotoxicity in children following paracetamol intoxication, particularly following *multiple sequential overdoses*, and that the mortality in these cases can be high. Factors reported as increasing the risk of liver injury and hepatotoxicity includes delayed referral and/or management and concomitant ingestion of other hepatotoxic agents [56, 57].

Evidence from metabolism studies, case reports and larger systematic retrospective case reviews but no prospective controlled trials demonstrate that following *single acute paracetamol overdose*, children may be relatively protected from acute liver injury and hepatotoxicity when compared to adults [15, 46, 51–53, 58]. However, following *multiple overdoses* which is the predominant mode of paediatric overdose has been reported [15, 51, 56–58]. Factors conferring relative protection to children from the toxic effect of paracetamol overdose include increases in paracetamol sulphation capacity [44–48], relative immaturity of the CYP450 oxidation system [15, 46, 49, 51] and the larger relative liver volume found in children [59] but these theoretical mechanisms have not been confirmed.

#### Older people

A number of investigators have looked into the effect of advancing age on the metabolism of paracetamol and whether this increases the likelihood of paracetamol-induced toxicity. Two large population-based studies reported that the rate of paracetamol poisoning decreased with age from a peak in adolescence and early adulthood [60, 61].

The data related to paracetamol metabolism and age are inconsistent despite there being a number of investigations published (> 150 subjects) [61–72]. Some PK studies report an increased theoretical risk of toxicity with advancing age [62–67] due to prolongation of t_1/2_ [62, 63], reduced paracetamol clearance [64, 67], increased paracetamol oxidation caused by a reduction in glucuronidation and sulphation capacity [65] or a reduction in liver volume [66]. Conversely, other PK studies imply that increasing age has no effect on toxicity [68–72]. These studies report that paracetamol t_1/2_ [68, 69, 71], AUC and oral clearance [71] are unrelated to age, that the rate of recovery of the products of paracetamol oxidative metabolism is not affected in older subjects [69], that the rate of conjugation is also not affected by increasing age [69, 70] and that a higher *C*_max_ in older people does not translate into changes in ALT [72]. A retrospective case series reported that, although the frequency of paracetamol poisoning is lower in older subjects, when paracetamol intoxication does occur, it is more frequently associated with fulminant hepatic failure and death and age is considered an independent risk factor of morbidity and mortality in these cases [61].

These studies demonstrate inconsistent findings and this inconsistency probably represents the heterogeneity of older people in terms of co-morbidities, liver volume, organ dysfunction and concurrent medications. Differences in experimental design are also likely to contribute to differences in results. It remains unclear whether any changes in PK translate to an increased propensity to toxicity with no studies demonstrating this conclusively. Clinical experience suggests that many older people do take regular paracetamol without development of toxicity. There is a lack of good quality clinical evidence that older people have a clinically significant difference in paracetamol metabolism or are at increased risk of toxicity at either therapeutic or supra-therapeutic doses.

### Nutritional state and body weight

It has been suggested that acute and chronic malnutrition may give rise to an increased propensity to paracetamol toxicity, especially if multiple concurrent doses are taken [73, 74]. Malnutrition was an indication for using the high-risk treatment line when two treatment lines were used to determine need for NAC treatment in the UK [75]. It was believed that a malnourished state depleted hepatic GSH and thus reduced the ability to detoxify NAPQI leading to increased risk of toxicity [73, 76]. Moreover, it has been suggested that starvation reduces liver glycogen storage and hence reduces UGT conjugative ability leading to a greater fraction of the dose being oxidised to NAPQI [77]. These conclusions came from the observation of unexpected liver injury/hepatotoxicity, often as case reports, in patients clinically assessed as being ‘malnourished’ [73, 78–81]. However, malnutrition and starvation are difficult to assess clinically and in particular protein-calorie malnutrition can co-exist with chronic alcohol use and with inter-current acute or chronic illness, which may independently or in concert increase the risk of paracetamol toxicity and confound the assertion that malnutrition per se increases the risk of paracetamol toxicity [6, 16, 73, 78–83].

The observation that causality has not been proven has led some authors to cast doubt about the role of malnutrition as a risk factor for paracetamol toxicity; indeed there is no convincing evidence that malnutrition does increase the risk. (see Table 6 in the supplementary data for a summary of the evidence) [6, 16, 82–84].

In protein-calorie malnutrition, both GSH and CYP2E1 quantities would be reduced (as detailed by a reduction in both GSH and CYP2E1 messenger RNA) and thus there would be no net overall effect on toxicity [16, 85]. A different paper suggests that there is no evidence that reduced food intake over the course of a few days reduces liver glutathione [82]. CYP2E1 in addition to metabolising paracetamol also metabolises acetone to glucose; it has been proposed that starvation increases ketone body formation and that these would compete with paracetamol for CYP2E1 and may potentially ‘reduce toxicity’ [82].

In the UK, the licenced oral paracetamol dose in adults is 4 g/day in 1 g doses every 4–6 h [86]. The same dose is recommended in the USA and in Australia, except that in the USA, the oral dose has been reduced to 350 mg per dosage unit in prescribed preparations [87, 88]. For simplicity, the recommended oral dose for children in the UK is in age bands; alternatively, 15 mg/kg is also recommended [86]. The UK regulator recommends that in adults weighing less than 50 kg, ‘clinical judgement’ should be employed when prescribing oral paracetamol as these patients may be at increased ‘risk of hepatotoxicity’ but does not specify a dose reduction. No recommendation for an oral dose reduction due to low body weight in the USA or Australia could be found. By contrast, for the intravenous (IV) preparation, the UK licence recommends a dose of 4 g/day every 4–6 h for adults weighing above 50 kg with a dose reduction to 3 g/day in those who have ‘risk of hepatotoxicity’. For adults weighing below 50 kg, the dose of IV paracetamol is recommended at 15 mg/kg every 4–6 h with a maximum dose of 60 mg/kg/day. The IV guidance is similar to that recommended in the USA and Australia.

There are multiple reports detailing cases of iatrogenic overdose of IV paracetamol in both children and adults. An iatrogenic IV overdose of paracetamol was the subject of a Scottish fatal accident inquiry [89, 90]. These case reports generally involve the overdose of subjects weighing less than 50 kg and the majority of these case reports concern dose miscalculation in paediatrics. Case reports exist of underweight individuals (< 50 kg) developing liver injury or hepatotoxicity when given ‘therapeutic doses’ of oral paracetamol [91, 92]. In these cases, individuals were given 133, 91 and 88 mg/kg/day orally over a number of days. While the doses were within the recommended range for oral administration, each patient was given more than the 60 mg/kg/day of paracetamol recommended for IV use but less than the 150-mg/kg/day limit recommended for immediate treatment of therapeutic excess. Interestingly, a multi-centre, randomised, double-blind, active controlled, parallel group trial of long-term paracetamol with naproxen as a comparator group for pain related to osteoarthritis over a 6–12-month period included patients in the weight range of 41–190 kg, with the lowest weight patient receiving 95.2 mg/kg/day [93]. Over the study period, there were no reports of hepatic dysfunction or failure.

Notwithstanding, the appropriate oral paracetamol dose in adults weighing less than 50 kg has not been investigated with liver injury or hepatotoxicity as a primary endpoint. In a review article describing toxicity from therapeutic doses of paracetamol, the weight of the patients was not considered in relation to development of toxicity and it would be informative to establish if the reports included in the review represented patients with a weight less than 50 kg and what the milligram/kilogram dose was [7].

Although there is no good-quality evidence to suggest that the dose of oral paracetamol should be reduced for individuals weighing less than 50 kg, it seems illogical that the oral recommendations differ from those for intravenous administration or the oral milligram/kilogram dose recommended for children. Indeed, some UK National Health Service organisations have independently implemented guidance suggesting that a lower dose of oral paracetamol should be prescribed in those weighing under 50 kg but this represents organisational cautiousness [94].

It is difficult to assess the contribution of weight and nutrition to paracetamol toxicity due to lack of good quality clinical evidence.

### Alcohol use

Case reports and retrospective case series have suggested that chronic alcohol consumption increases the risk of toxicity from paracetamol, perhaps even with therapeutic drug doses [95–108]. A number of case reports describe patients developing acute liver injury and hepatotoxicity from a therapeutic dose and conclude that patients with chronic alcohol use are at heightened risk of paracetamol toxicity [95, 96, 106, 108, 109]. However, during alcohol consumption, the sensorium may be clouded making the reliability of the dose history uncertain. Due to concerns around increased vulnerability to paracetamol toxicity, chronic alcohol consumption was a reason to use the high-risk treatment link for consideration of NAC treatment when risk stratification was recommended [75].

It has been suggested that chronic alcohol abuse increases susceptibility to paracetamol toxicity due to CYP2E1 induction [95, 96, 98–101], that hepatic GSH is reduced leading to reduced NAPQI detoxification [95, 96, 98–101, 103, 104, 106, 109] and/or that glucuronidation is reduced leading to increased fractional oxidation [95–97]. Other theories advanced for the observation of increased toxicity in chronic alcohol consumers include disturbances to hepatocyte membranes rendering them more vulnerable to insult, decreased biliary excretion of paracetamol or reduced clearance [97]. In a controlled study investigating the effect of ethanol infusion on paracetamol metabolism, subjects exposed to ethanol were found to have an increase in the recovery of the oxidative products of paracetamol metabolism compared to controls but only by a small percentage (22%), and increased oxidation was detected 8 h after the ethanol infusion had ceased [110]. By contrast, acute alcohol co-ingestion with paracetamol may reduce the risk of toxicity because alcohol competes for CYP2E1 and prevents paracetamol metabolic activation [16, 20, 102, 109, 111–117]. However, prospective studies done in controlled circumstances or systematic reviews have found no association with chronic alcohol use and increased susceptibility to paracetamol toxicity at therapeutic doses [16, 84, 100, 113, 116–118].

Some authors have suggested that, once established, paracetamol toxicity may be more severe in those with chronic alcohol consumption [119] although this is not a universal finding [118]. This may be because chronic alcohol abusers often present late with therapeutic misadventure, when toxicity is established [83, 95–99].

**Fig. 3:**
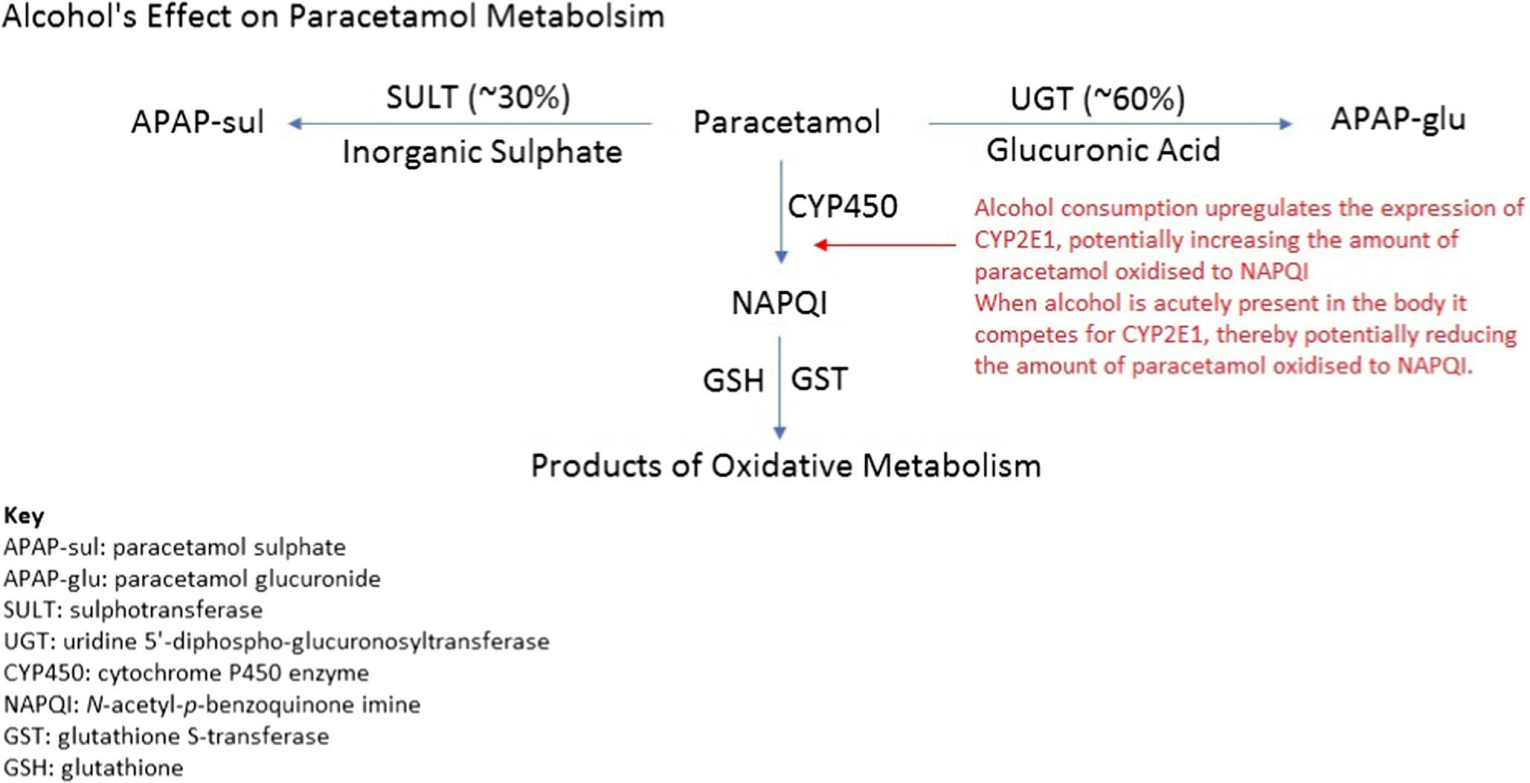
Alcohol’s effect on paracetamol metabolism

There is no good quality clinical evidence from prospective trials that alcohol consumption increases the risk of paracetamol toxicity (see Table 6 in the supplementary data for a summary of the evidence and Fig. 3 for alcohol’s effects on paracetamol metabolism).

### Potential drug interactions with paracetamol metabolism

**Fig. 4:**
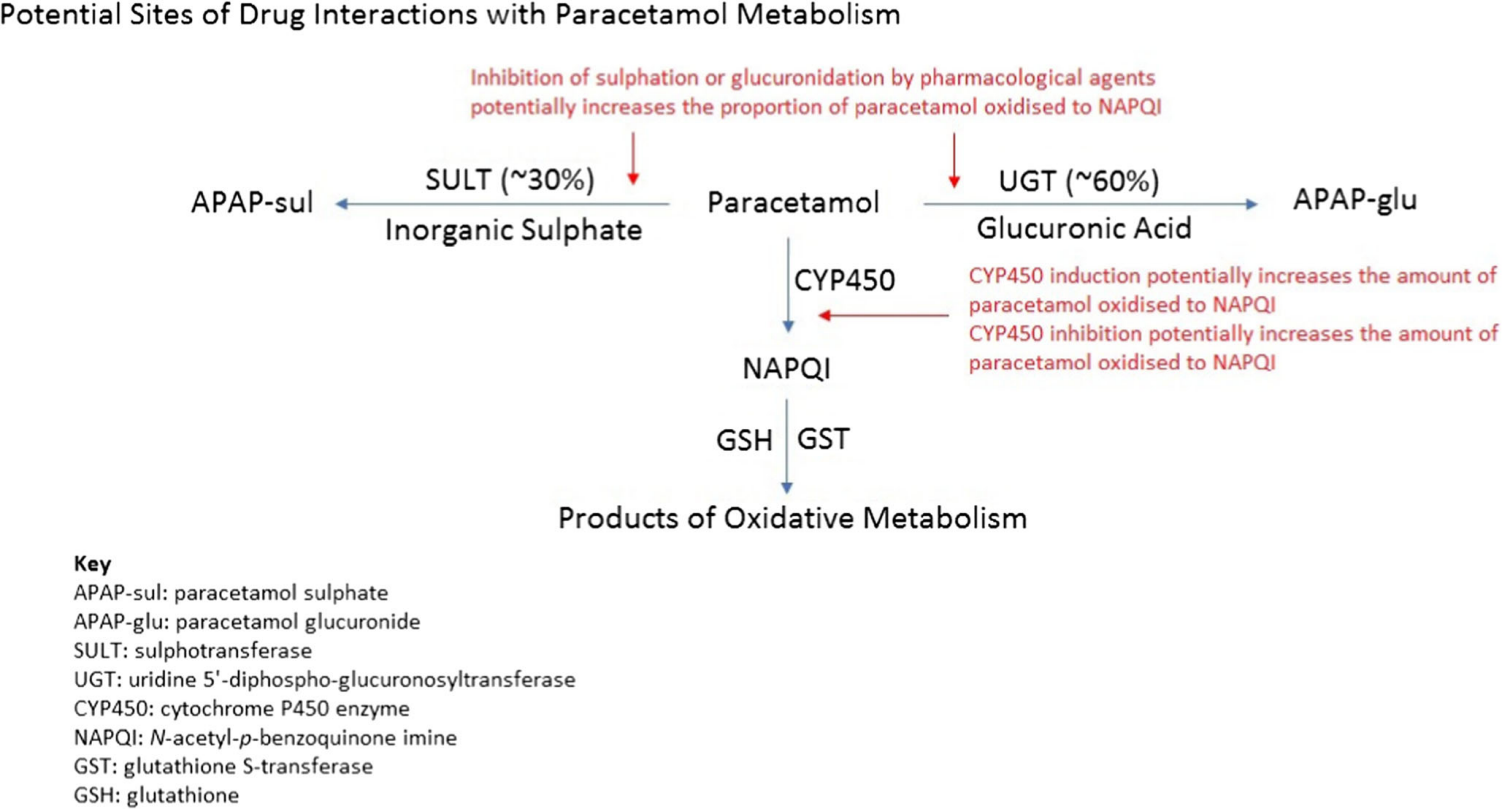
Potential sites of drug interactions with paracetamol metabolism

Many case reports state that certain pharmacological agents affect the metabolism of paracetamol, especially in relation to rendering patients more susceptible to toxicity. Putative mechanisms include enzyme induction (increased paracetamol oxidation), enzyme inhibition (reduced conjugation) and glutathione depletion (reduced NAPQI detoxification) (see Fig. 4 for potential sites of drug interactions with paracetamol metabolism). However, aside from case reports, there is very little evidence that drug interactions increase the risk of liver injury.

### Enzyme induction

It has been proposed that pharmacological agents that induce the CYP450 enzymes responsible for paracetamol oxidation could theoretically increase the fraction of paracetamol oxidised to NAPQI. A literature review concludes that therapeutic doses of paracetamol with or without CYP450 enzyme inducers do not lead to an increased propensity to toxicity [82]. See Tables 8, 9, 10, 11, 12, and 13 in the supplementary data for a summary of the evidence relating to paracetamol hepatotoxicity and exposure to CYP450 enzyme inducers.

### Enzyme inhibition

It is postulated that drugs that inhibit glucuronidation may increase the fractional proportion of paracetamol shunted to oxidation. There is no good evidence that co-administration of drugs that inhibit glucuronidation increases an individual’s risk of paracetamol toxicity. See Tables 14, 15, 16, 17, 18 in the supplementary data for a summary of the evidence relating the enzyme-inhibiting drugs and paracetamol toxicity.

### Reduced levels of glutathione

GSH binds NAPQI, thereby detoxifying it [120]. Disease states may reduce the level of hepatic glutathione. A recent literature review suggests that there is no convincing evidence that these increase the risk of paracetamol toxicity at therapeutic doses [84].

### Paracetamol and chronic liver disease

Due to its ability to cause liver toxicity in supra-therapeutic doses, there is theoretical concern about the administration of paracetamol to patients with chronic liver disease (CLD).

Studies have demonstrated that there is not an increase in CYP450 activity in CLD but rather that the enzyme activity remains unchanged or decreases [121, 122]. Moreover, although there is variation in CYP450 activity among both healthy and diseased livers, cirrhosis was associated with a significant decrease in CYP2E1 activity [123].

Investigation into the concentration of hepatic and plasma GSH in liver disease has been mixed, with some reporting a decrease in glutathione [124, 125] and others reporting an increase [126, 127]. Even if GSH levels are decreased in CLD, they would not be decreased to such an extent as to cause toxicity at therapeutic doses as the studies have demonstrated that chronically diseased livers remain capable of producing GSH [76, 120].

The t_1/2_ of paracetamol is known to be increased in patients with toxicity secondary to paracetamol overdose [128, 129], viral hepatitis [130] and in patients with CLD [131–136]. However, in stable chronic liver disease, there is no accumulation of paracetamol [134] and the excretion of toxic metabolites remained the same between those with mild or severe liver disease and controls [131].

The UK regulator does not recommend a dose reduction in hepatic disease but suggests avoiding large doses [86]. On the basis of records in the US Federal Drug Administration’s Adverse Events Reporting System and the Multiple Causes of Death Files, which may signal a disproportionate prevalence of patients with pre-existing liver disease developing liver injury when using paracetamol, but not on the basis of conclusive evidence, the US currently requires paracetamol-containing products to be labelled ‘ask a doctor before using [paracetamol] if you have liver disease’, but stops short of recommending a dose alteration [137]. It is likely that the therapeutic doses of paracetamol are safe to use in both adults and children with CLD; no studies have been conducted into the administration of paracetamol during acute liver disease.

## Conclusions

Given that paracetamol is one of the most widely consumed medications globally, it is perhaps surprising that there are few quality data that inform whether certain individuals have a greater propensity to develop liver injury than others. Although observational data suggest that non-overdose paracetamol ingestion may cause liver failure in a very small proportion of people and ALT rises of uncertain clinical significance occur in some patients treated with paracetamol in RCTs, there is no good evidence that *therapeutic* doses of paracetamol present a greater risk of toxicity in any group covered by this review [24–26].

A key question is does this lack of robust data matter? Given its widespread use, any small increase in risk of toxicity could translate into vulnerable individuals being harmed. As the efficacy of paracetamol has been questioned in the setting of chronic pain, it is important to understand safety/toxicity, in order to protect users from unacceptable harms.

In our view, there are two particular scenarios that warrant further discussion and research. The first setting is a speculative future challenge. It is becoming increasingly cheap and straightforward to generate genetic information on people, including whole genome sequencing. In the near future, it is possible that a patient’s genomic information will be available as a tool to guide prescribing within the context of *precision medicine.* If, or when, this situation is a reality, we may still not understand whether the patient’s genome has a clinically important effect on paracetamol metabolism and risk of toxicity. This highlights an important disconnection between advancements in genomic medicine and our understanding of the clinical phenotype induced by gene changes with regard to both pharmacokinetics and pharmacodynamics or the role of genetic variation in the body’s response to liver injury when it does occur. However, any future understanding of genomics altering paracetamol metabolism and thus liver injury or altering the body’s response to such an injury is unlikely to affect the vast majority of patients who present following overdose, as pre-clinical data suggests that everyone would develop liver injury given ‘sufficiently high’ doses. In this future setting, there would be an argument for robustly defining the risk of paracetamol with regard to genetics and especially whether ‘sufficiently high’ dose to cause liver injury in some patients falls within or just above the therapeutic range. Failure to do this could lead to confusion among patients and doctors, as genetic information will be available without clarity about its impact on paracetamol prescribing and overdose management.

At present, there is persisting confusion about whether the oral dose of paracetamol should be reduced in adults with low body weight. This has resulted in individual healthcare providers recommending dose reductions that are not reflected in the drug licence. This leads to conflicting messages about what constitutes an overdose. For example, a hospital may recommend dose reduction in a patient weighing less than 50 kg but the same patient would take a higher dose paracetamol if they used the drug packaging for dosage information in the community. We would suggest that research is needed in this area to identify safety signals in low body weight, but otherwise healthy, individuals. Such research could incorporate novel biomarkers of liver injury in mechanistic studies and population level data linkage.

## Electronic supplementary material

ESM 1 (DOCX 91 kb)
