# Supplementary material for: Are some people at increased risk of paracetamol-induced liver injury? A critical review of the literature
Source: Eur J Clin Pharmacol. 2017 Oct 24;74(2):147–60. doi: 10.1007/s00228-017-2356-6 (PMC5765191; doi:10.1007/s00228-017-2356-6)
Supplement: Supplementary file 1 — (DOCX 91 kb) [file 228_2017_2356_MOESM1_ESM.docx]

# Supplementary Data Document

**EJCP, Caparrotta, Antoine & Dear -** EJCL-D-17-00516

| Sulfotransferase Activity - Unclear risk after an overdose |
| --- |
| - 29 subjects were given paracetamol to ascertain whether variation in platelet sulphotransferase activity correlated with APAP-sul excretion; significant correlation was demonstrated; toxic metabolites were not measured.^140^ |
| - 13 volunteers given paracetamol demonstrated a wide inter-individual variation in paracetamol metabolism; no correlation between platelet sulphotransferase activity and the excretion of paracetamol metabolites.^141^ |

Table 1: Summary of evidence of alterations in paracetamol metabolism due to sulphotransferase activity

| CYP2E1 Polymorphisms - Unclear risk after an overdose |
| --- |
| - 15 patients with alcoholic liver disease investigating mutant CYP2E1 *RSaI/PStI* polymorphism; individuals in possession of the homozygous mutant polymorphism had a shorter paracetamol elimination t_1/2_ compared to homozygous wildtype individuals suggesting a higher activity of mutant CYP2E1; study was small; paracetamol metabolites were not quantified; significance unclear.^142^ |

Table 2: Summary of evidence of CYP2E1 polymorphisms affecting paracetamol metabolism

| CYP2D6 Copy Number Variation - Theoretical increased risk before/after an overdose |
| --- |
| - CYP2D6 metabolises paracetamol at ~33% of the efficiency of CYP2E1; contribution of CYP2D6 may be greater in ultra-rapid extensive metabolisers (CYP2D6 gene duplication); *in vitro* study; paracetamol metabolites not quantified; significance unclear.^143^ |

Table 3: Summary of evidence of CYP2D6 polymorphisms affecting paracetamol metabolism

| GST Polymorphisms - Theoretical increased risk after an overdose |
| --- |
| 104 paracetamol poisoned patients were genotyped retrospectively without controls to explore the effect of GSTT1, GSTM1 and GSTP1 polymorphisms in humans; borderline association was found between the GSTT1 homozygous deletion genotype and a high trough prothrombin time (a marker of severity of paracetamol poisoning) compared to those carrying two functional alleles. The frequency of the GSTP1 Val/Val genotype was significantly lower in poisoned patients than in the background population. GSTT1 homozygous deletion genotype may be associated with a better prognosis following paracetamol poisoning and the GSTP1 homozygous variant genotype may confer a decreased risk of paracetamol poisoning.^144^ There is no evidence of attempts to reproduce these findings by other investigators. |

Table 4: Summary of evidence of GSH polymorphisms affecting paracetamol metabolism

| Inter-individual Variation and Ethnicity - Theoretical increased risk after an overdose |
| --- |
| - 111 Scottish Caucasians, 67 Ghanaians and 20 Kenyans; 24-hours urinary metabolites of 1.5g paracetamol; GSH-derived metabolites were high in Caucasians compared to the other groups due to a relative reduction in glucuronidation. Authors speculate that Caucasians may be at higher risk of toxicity from paracetamol but offer no direct evidence to support this. ^28^ |
| - 39 Basque subjects and 32 subjects from Alicante found no difference in the recovery of the oxidative products of metabolism between these groups but report that the rate of oxidation was intermediate between Africans and Caucasians reported in ^28^. Authors consider that the Spanish populations may be at higher risk of paracetamol toxicity on the basis of this difference but offer no conclusive evidence to back up this assertion.^28,145^ |
| - 145 healthy individuals; five group, single-blind, randomised placebo-controlled study, modified intent-to-treat analysis examined paracetamol alone or paracetamol in combination with opiates (1g paracetamol four times/day); significant rise in ALT in Hispanic patients compared to other ethnicities with all ALT levels in the paracetamol-induced ALI range and no liver toxicity noted; no other evidence of toxicity. Not stated why this may be the case but authors note studies where ethnic differences in metabolism are reported. Those of Hispanic origin may have a greater propensity to fatty liver disease which authors conjecture may increase the risk of paracetamol toxicity; no clinical significance attached to rise in ALT detected.^25^ |
| Theoretical risk reduction after an overdose |
| - 20 healthy patients in Hong Kong given 20mg/kg paracetamol; 11 of Chinese ethnicity and nine Caucasians. APAP-glu and products of oxidative metabolism were lower and the APAP-sul concentration was higher in Chinese subjects who may be at lower risk of paracetamol liver injury than Caucasians by extrapolation of these findings; no conclusive evidence to support this.^146^ |
| - A retrospective, observational study examined 224 Chinese patients admitted with paracetamol poisoning to a Hong Kong hospital. 28 patients were deemed to be at risk of toxicity; 13 *developed* acute liver injury; five were assessed as having hepatotoxicity; no control patients of another ethnicity and all recovered with treatment with NAC. Authors conjecture, lower rate of hepatotoxicity (and renotoxicity) is due to low rates of alcohol consumption and ethnic differences; authors conclude that those of Chinese ethnicity are at lower risk of hepatotoxicity by observing that the rate of hepatotoxicity is lower than that reported in other studies.^147^ Conclusions speculative. |
| Theoretical no change in risk after an overdose |
| - 12 healthy Caucasians and 12 Chinese subjects in Australia following ingestion 1g of paracetamol found no difference in the rate of urinary recovery of the products of paracetamol oxidation.^148^ |
| - No difference in the rate of oxidative metabolism found between six Chinese patients taking phenytoin, six taking carbamazepine and 20 healthy controls given 20mg/kg of paracetamol; ratio of oxidative metabolite excretion was found to be similar to that reported in Scottish Caucasians in ^28^.^149^ |
| - 24 Chinese and 24 Indians subjects living in Singapore given 15mg/kg of paracetamol also reported that the recovery of the urinary metabolites was no different between the groups but they had a significantly lower rate of oxidative metabolism when compared to data reported in another paper ^28^ describing the excretion of oxidative metabolites in Caucasians in Scotland.^150^ |

Table 5: Summary of evidence that ethnicity affects paracetamol metabolism and toxicity

| Nutritional State - Clinical evidence of increased risk of toxicity |
| --- |
| - A cohort study looking at paracetamol toxicity suggests that fasting may be a risk factor for paracetamol-induced toxicity but all patients who developed derangement in their liver function took more than the recommended 4g/day dose of paracetamol.^75^ |
| - Case reports which detail cases of paracetamol toxicity within the therapeutic range and implicate fasting as the primary reason for the development of liver dysfunction feature individuals with other putative risk factors for liver injury such as malignancy, recent general anaesthetic, chronic alcohol excess or coinfection with human immunodeficiency virus, hepatitis C or campylobacter, and these may confound their conclusions.^6,80,82,83^ |
| Nutritional State - Theoretical no change in risk after an overdose |
| - Study investigating the effect of fasting on paracetamol metabolism in controlled conditions found only small amounts of paracetamol oxidation conjugates in the urine, albeit in circumstances where the studied population was overweight and only half the daily maximum dose was administered.^151^ |

Table 6: Selection of evidence regarding the differences of paracetamol metabolism and propensity to toxicity due to nutritional state

| Alcohol Use - Theoretical increased risk an overdose |
| --- |
| - Eight male alcohol-dependent subjects were found to have significantly lower blood GSH concentrations than healthy volunteers (4.35µM vs. 8.48 µM); plasma GSH concentrations fell further following paracetamol ingestion (2.4 µM vs. 6.26 µM).^106^ |
| - 10 healthy volunteers received a six-hour ethanol infusion or 5% dextrose; 500mg paracetamol administered eight-hours post-infusion. NAPQI metabolites increased by 22% in those who has been administered ethanol infusion but only once alcohol has been cleared from the body.^112^ |
| Alcohol Use - Theoretical reduced risk after an overdose |
| - 10 subjects, five heavy drinkers, 5 occasional drinkers given 20mg/kg paracetamol. Significant decreased in NAPQI metabolites in heavy drinkers.^116^ |
| Clinical evidence that alcohol consumption increases the risk of paracetamol toxicity |
| - Multiple case reports/series describing acute livery injury and hepatotoxicity following therapeutic doses of paracetamol in chronic alcohol-dependents.^97–110^ |
| - Case series: 67 patients, 64% >80g alcohol/day, 35% ≤60g alcohol/day, remainder vague alcohol history. All developed hepatotoxicity with participants reporting <6g paracetamol intake per day.^97^ |
| - Retrospective cohort study: 71 patients, chronic alcohol abuse associated with accidental overdose, acute alcohol ingestion associated with accidental and deliberate overdose. Chronic alcohol use increased severity of liver toxicity following overdose. No synergy between paracetamol and alcohol established.^85^ |
| - 209 consecutive patients presenting with a single, acute paracetamol overdose. Chronic alcohol consumption significantly and independently associated with increased risk of hepatic coma, lower PT index, lower platelets, higher creatinine and higher bilirubin. Acute alcohol ingestions not significantly associated with any dependent variables.^121^ |
| Clinical evidence that alcohol consumption does not increase the risk of paracetamol toxicity |
| - Double-blind, randomised placebo-controlled trial of 102 patients given paracetamol orally 4g/day and 99 patients given placebo, all alcohol-dependent; repeated doses of paracetamol at maximum daily dose to alcohol-dependents does not increase risk of toxicity.^115^ |
| - Systematic review: good quality data does not suggest that repeated administration of therapeutic doses of paracetamol to alcohol-dependents increases the risk of toxicity.^119^ |

Table 7: summary of key evidence relating to alcohol ingestion and paracetamol metabolism and toxicity

#### Carbamazepine and Paracetamol Toxicity

| Pharmacokinetic Studies | - Mixed results. - One study suggests an increase in NAPQI formation.^152^ - Other studies do not demonstrate an increase in NAPQI formation.^149,153,154^ |
| --- | --- |
| Clinical studies | - Multiple case reports suggesting that carbamazepine potentiates toxic effects of paracetamol.^155–158^ |
| Other studies | - Carbamazepine induces CYP450, but is itself hepatotoxic.^88^ - There is no evidence to suggest that carbamazepine induces CYP450 in humans.^159^ - It is unlikely that carbamazepine enhances paracetamol toxicity.^62^ |
| Conclusions | - No good evidence that carbamazepine increases paracetamol toxicity. |

Table 8: Evidence summary of carbamazepine's effect on paracetamol metabolism and toxicity

#### Phenytoin and Paracetamol Toxicity

| Pharmacokinetic Studies | - Mixed results. - Metabolite/paracetamol ratio and GSH-derived metabolites increased in patients given phenytoin and paracetamol concurrently; no data on liver toxicity.^160^ - PK studies suggest that phenytoin has no effect on the formation of NAPQI metabolites.^153,153,154^ - Phenytoin has a protective effect, less NAPQI metabolite was detected in patients taking phenytoin than patients not exposed to it.^149^ |
| --- | --- |
| Pre-clinical studies | - Studies in cultured human hepatocytes suggest that co-administration of phenobarbital with phenytoin may increase oxidative metabolism by reducing glucuronidation but only when present simultaneously.^161^ |
| Clinical studies | - Multiple case reports suggest that phenytoin potentiates the toxic effects of paracetamol; often in patients taking mixed anti-epileptics or following massive overdoses.^162–165^ - Longer term data suggest that taking ‘anticonvulsant’ therapy does not increase the risk of liver toxicity following overdose.^62^ |
| Other studies | - Review article: phenytoin does not induce CYP2E1 but may be hepatoprotective in overdose.^84^ |
| Conclusion | - No good evidence that phenytoin increases paracetamol toxicity, indeed it may be hepatoprotective. |

Table 9: Evidence summary of phenytoin's effect on paracetamol metabolism and toxicity

#### Rifampicin and Paracetamol Toxicity

| Pharmacokinetic Studies | - Controlled study: rifampicin was not demonstrated to increase the toxic metabolites of paracetamol; indeed it was shown to increase glucuronidation and clearance and thus may reduce paracetamol’s analgesic effects.^153,166^ |
| --- | --- |
| Clinical studies | - Case reports have drawn attention to a potential association of increased liver toxicity in patients being concomitantly treated with rifampicin.^167,168^ |
| Other studies | - Rifampicin does not induce CYP2E1.^166^ - Systematic review article: It appears unlikely that rifampicin increases the risk of paracetamol toxicity.^122^ |
| Conclusion | - No good evidence that rifampicin increases paracetamol toxicity. |

Table 10: Evidence summary of rifampicin's effect on paracetamol metabolism and toxicity

#### Isoniazid and Paracetamol Toxicity

| Clinical studies | - Case reports have suggested that due to CYP450 induction, isoniazid may potentiate the risk of paracetamol liver toxicity.^168–170^ - Formal studies demonstrated that, while CYP2E1 is induced by isoniazid ^171^ its presence prevents paracetamol oxidation by competition and leads to a smaller fraction of the paracetamol dose being oxidised.^171,172^ - NAPQI formation was only increased in those with CYP2E1 fast-acetylator phenotype following cessation of isoniazid treatment and then only by a small percentage unlikely to be of toxicological significance.^172^ - A study looking at liver injury in those on long term tuberculosis treatment found no evidence of toxicity in those receiving concomitant isoniazid and paracetamol.^173^ |
| --- | --- |
| Other studies | - Isoniazid is known to induce CYP2E1.^19,172,174^ |
| Conclusion | - No good evidence that isoniazid increases paracetamol toxicity. |

Table 11: Evidence summary of isoniazid's effect on paracetamol metabolism and toxicity

#### Omeprazole and Paracetamol Toxicity

| Pharmacokinetic Studies | - Studies looking at CYP450 enzyme induction suggest that omeprazole has the potential to increase NAPQI formation by inducing CYP1A2, although it does not induce CYP2E1.^175,176^ |
| --- | --- |
| Clinical Studies | - No case reports that associate omeprazole with paracetamol toxicity. - Controlled studies in humans do not demonstrate an increase in the formation of paracetamol toxic metabolites.^20,177^ |
| Conclusion | - There is no good evidence that omeprazole increases paracetamol toxicity. |

Table 12: Evidence summary of omeprazole's effect on paracetamol metabolism and toxicity

#### Tobacco Consumption and Paracetamol Toxicity

| Pharmacokinetic Studies | - Smoking tobacco may induce CYP2E1.^178^ - Studies have suggested that tobacco smoking increases the rate of first pass metabolism of paracetamol ^13,179^ by increases in glucuronidation.^179^ - Smoking may also increase the rate of paracetamol clearance.^28^ |
| --- | --- |
| Clinical Studies | - No reports of tobacco smoking being associated with paracetamol toxicity. - Following overdose, tobacco use has been found to be an independent predictor or mortality although this may not be due to altered paracetamol metabolism but rather via other effects.^180^ - Systematic review article: overall smoking has demonstrated no consistent effect on paracetamol metabolism.^181^ |
| Conclusion | - There is no good evidence that tobacco consumption increases paracetamol toxicity. |

Table 13: Evidence summary of tobacco consumption's effect on paracetamol metabolism and toxicity

#### H_2_ Receptor Antagonists and Paracetamol Toxicity

| Pharmacokinetic Studies | - Metabolic studies have suggested that ranitidine increases paracetamol AUC by inhibiting glucuronidation ^182^ although another similar study demonstrated no change in paracetamol metabolism.^183^ - Studies suggested that cimetidine does not affect the conversion of paracetamol to its toxic metabolites.^116,184^ - Another study reports that cimetidine decreased the amount of toxic metabolite cleared compared to non-toxic metabolites.^185^ - Further investigations suggest that cimetidine has no effect paracetamol pharmacokinetics or metabolite formation ^186–188^, however others suggest that cimetidine shortens t_1/2_, reduces C_max_ and increases time to maximum plasma concentrations due to first bypass metabolism. |
| --- | --- |
| Clinical Studies | - Ranitidine has been associated with paracetamol toxicity when co-administered with paracetamol in a case report.^189^ - The addition of cimetidine to NAC when treating paracetamol overdose did not confer a hepatoprotective effect.^190^ |
| Conclusion | - There is no good evidence that H_2_ receptor antagonist consumption increases or decreases paracetamol toxicity nor that it affects paracetamol’s efficacy. |

Table 14: Evidence summary of H2 receptor antagonists’ effect on paracetamol metabolism and toxicity

#### Disulfiram and Paracetamol Toxicity

| Pharmacokinetic Studies | - Disulfiram is metabolised to diethycarbamate, a potent inhibitor of CYP2E1.^184^ - Two studies suggest that disulfiram reduces NAPQI formation if paracetamol is administered after disulfiram administration.^19,184^ - One study suggests that disulfiram has no effect on the partial clearance of paracetamol metabolites including NAPQI metabolites.^191^ |
| --- | --- |
| Clinical Studies | - No case reports to suggest hepatoprotection. - No controlled clinical trials. |
| Conclusion | - There is no good evidence that disulfiram consumption decreases paracetamol toxicity. |

Table 15: Evidence summary of disulfiram's effect on paracetamol metabolism and toxicity

#### Tyrosine Kinase Inhibitors and Paracetamol Toxicity

| Pharmacokinetic Studies | - Study *in vitro* suggests that sorafenib, dasatinib and imatinib reduce paracetamol glucuronidation, potentially increasing the fraction of the drug oxidised to NAPQI.^192^ - Controlled investigation in humans suggests that the pharmacokinetics and metabolites of paracetamol are not different to controls not treated with imatinib.^193^ |
| --- | --- |
| Clinical Studies | - Case report exists describing toxicity in a patient taking sunitinib and paracetamol concurrently.^194^ - No controlled clinical trials. |
| Conclusion | - There is no good evidence that tyrosine kinase inhibitors decrease or increase paracetamol toxicity. |

Table 16: Evidence summary of tyrosine kinase inhibitors' effect on paracetamol metabolism and toxicity

#### Zidovudine and Paracetamol Toxicity

| Pharmacokinetic Studies | - A study into the pharmacokinetics of paracetamol and zidovudine, found no change in paracetamol concentration.^195^ |
| --- | --- |
| Clinical Studies | - Toxicity associated with zidovudine and paracetamol co-administration has been described in a case report. Zidovudine is known to be hepatotoxic of itself. The postulated mechanism of increased toxicity is by decreased paracetamol glucuronidation leading to increased oxidation.^196^ - No controlled clinical trials. |
| Conclusion | - There is no good evidence that zidovudine decreases or increases paracetamol toxicity. |

Table 17: Evidence summary of zidovudine's effect on paracetamol metabolism and toxicity

#### Phenobarbital and Paracetamol Toxicity

| Pharmacokinetic Studies | - Co-administration with sub-toxic doses of phenobarbital lead to increased recovery of the products of oxidation in humans by suppressing glucuronidation of paracetamol.^197^ |
| --- | --- |
| Clinical Studies | - A case report of toxicity associated with co-administration of paracetamol and phenobarbital exists.^198^ - Case series, of whom five patients were on phenobarbital, reported worse toxicity than would be expected from other data; >8 hours from overdose, not given NAC.^165^ - No controlled clinical trials. |
| Conclusion | - There is no good evidence that phenobarbital increases the risk of paracetamol toxicity. |

Table 18: Evidence summary of phenobarbital's effect on paracetamol metabolism and toxicity

# References

1 Reiter C, Weinshilboum R. Platelet phenol sulfotransferase activity: Correlation with sulfate conjugation of acetaminophen. *Clinical Pharmacology & Therapeutics* 1982; **32**: 612–621.

2 Bonham Carter SM, Rein G, Glover V, Sandler M, Caldwell J. Human platelet phenolsulphotransferase M and P: substrate specificities and correlation with in vivo sulphoconjugation of paracetamol and salicylamide. *Br J Clin Pharmacol* 1983; **15**: 323–330.

3 Ueshima Y, Tsutsumi M, Takase S, Matsuda Y, Kawahara H. Acetaminophen Metabolism in Patients with Different Cytochrome P-4502E1 Genotypes. *Alcoholism: Clinical and Experimental Research* 1996; **20**: 25a–28a.

4 Dong H, Haining RL, Thummel KE, Rettie AE, Nelson SD. Involvement of Human Cytochrome P450 2D6 in the Bioactivation of Acetaminophen. *Drug Metab Dispos* 2000; **28**: 1397–1400.

5 Buchard A, Eefsen M, Semb S, Andersen SE, Morling N, Bendtsen F *et al.* The role of the glutathione S-transferase genes GSTT1, GSTM1, and GSTP1 in acetaminophen-poisoned patients. *Clinical Toxicology* 2012; **50**: 27–33.

6 Critchley JA, Nimmo GR, Gregson CA, Woolhouse NM, Prescott LF. Inter-subject and ethnic differences in paracetamol metabolism. *Br J Clin Pharmacol* 1986; **22**: 649–657.

7 Esteban A, Calvo R, Pérez-Mateo M. Paracetamol metabolism in two ethnically different Spanish populations. *European Journal of Drug Metabolism and Pharmacokinetics* 1996; **21**: 233–239.

8 Watkins PB, Kaplowitz N, Slattery JT, Colonese CR, Colucci SV, Stewart PW *et al.* Aminotransferase Elevations in Healthy Adults Receiving 4 Grams of Acetaminophen Daily: A Randomized Controlled Trial. *JAMA* 2006; **296**: 87–93.

9 Critchley JH, Critchley LH, Anderson PJ, Tomlinson B. Differences in the single-oral-dose pharmacokinetics and urinary excretion of paracetamol and its conjugates between Hong Kong Chinese and Caucasian subjects. *Journal of Clinical Pharmacy and Therapeutics* 2005; **30**: 179–184.

10 Chan T, Critchley J, Chan C. Renal failure is uncommon in Chinese patients with paracetamol (acetaminophen) poisoning. *Vet Hum Toxicol* 1995; **37**: 154–156.

11 Osborne NJ, Tonkin AL, Miners JO. Interethnic differences in drug glucuronidation: a comparison of paracetamol metabolism in Caucasians and Chinese. *Br J Clin Pharmacol* 1991; **32**: 765–767.

12 Tomlinson B, Young RP, Ng MCY, Anderson PJ, Kay R, Critchley JH. Selective liver enzyme induction by carbamazepine and phenytoin in Chinese epileptics. *E J Clin Pharmacol* 1996; **50**: 411–415.

13 Lee HS, Ti TY, Koh YK, Prescott LF. Paracetamol elimination in Chinese and Indians in Singapore. *European Journal of Clinical Pharmacology* 1992; **43**: 81–84.

14 Whitcomb DC, Block GD. Association of Acetaminophen Hepatotoxicity With Fasting and Ethanol Use. *JAMA* 1994; **272**: 1845–1850.

15 Kurtovic J, Riordan SM. Paracetamol-induced hepatotoxicity at recommended dosage. *Journal of internal medicine* 2003; **253**: 240–243.

16 Eriksson LS, Broome U, Kalin M, Lindholm M. Hepatotoxicity due to repeated intake of low doses of paracetamol. *Journal of internal medicine* 1992; **231**: 567–570.

17 Moling O, Cairon E, Rimenti G, Rizza F, Pristerá R, Mian P. Severe hepatotoxicity after therapeutic doses of acetaminophen. *Clinical Therapeutics* 2006; **28**: 755–760.

18 Vitols S. Paracetamol hepatotoxicity at therapeutic doses. *Journal of Internal Medicine* 2003; **253**: 95–98.

19 Schenker S, Speeg KV, Perez A, FINCH J. The effects of food restriction in man on hepatic metabolism of acetaminophen. *Clinical Nutrition* 2001; **20**: 145–150.

20 Lauterburg B, Velez M. Glutathione deficiency in alcoholics: risk factor for paracetamol hepatotoxicity. *Gut* 1988; **29**: 1153–1157.

21 Thummel KE, Slattery JT, Ro H, Chien JY, Nelson SD, Lown KE *et al.* Ethanol and production of the hepatotoxic metabolite of acetaminophen in healthy adults. *Clinical Pharmacology & Therapeutics* 2000; **67**: 591–599.

22 Critchley JAJH, Scott AW, Dyson EH, Jarvie DR, Prescott LF. Is there a place for cimetidine or ethanol in the treatment of paracetamol poisoning? *The Lancet* 1983; **321**: 1375–1376.

23 Zimmerman HJ, Maddrey WC. Acetaminophen (paracetamol) hepatotoxicity with regular intake of alcohol: analysis of instances of therapeutic misadventure. *Hepatology (Baltimore, Md)* 1995; **22**: 767–73.

24 Black M. Acetaminophen hepatotoxicity. *Annual review of medicine* 1984; **35**: 577–593.

25 Seeff LB, Cuccherini BA, Zimmerman HJ, Adler E, Benjamin SB. Acetaminophen Hepatotoxicity in Alcoholics: A Therapeutic Misadventure. *Annals of Internal Medicine* 1986; **104**: 399.

26 Wootton FT, Lee WM. Acetaminophen Hepatotoxicity in the Alcoholic. *Journal* 1990; **83**: 1047–1049.

27 Kumar S, Rex DK. Failure of Physicians to Recognize Acetaminophen Hepatotoxicity in Chronic Alcoholics. *Arch Intern Med* 1991; **151**: 1189–1191.

28 Riordan SM, Williams R. Alcohol exposure and paracetamol-induced hepatotoxicity. *Addiction Biology* 2002; **7**: 191–206.

29 Draganov P, Durrence H, Cox C, Reuben A. Alcohol-acetaminophen syndrome. *Postgraduate Medicine* 2000; **107**: 189–195.

30 Licht H, Seeff LB, Zimmerman HJ. Apparent potentiation of acetaminophen hepatotoxicity by alcohol. *Annals Of Internal Medicine* 1980; **92**: 511–511.

31 O’Dell JR, Zetterman RK, Burnett DA. Centrilobular Hepatic Fibrosis Following Acetaminophen-Induced Hepatic Necrosis in an Alcoholic. *JAMA* 1986; **255**: 2636–2637.

32 Lesser PB, Vietti MM, Clark WD. Lethal enhancement of therapeutic doses of acetaminophen by alcohol. *Digest Dis Sci* 1986; **31**: 103–105.

33 Florén C-H, Thesleff P, Nilsson Å. Severe Liver Damage Caused by Therapeutic Doses of Acetaminophen. *Acta Medica Scandinavica* 1987; **222**: 285–288.

34 Johnston SC, Pelletier LL. Enhanced hepatotoxicity of acetaminophen in the alcoholic patient. Two case reports and a review of the literature. *Medicine (Baltimore)* 1997; **76**: 185–191.

35 Emby BN, Fraser DJ&. Hepatotoxicity of paracetamol enchanced by ingestion of alcohol - report of two cases. *South African Medical Journal* 1977; **51**: 208–209.

36 Schiødt FV, Rochling FA, Casey DL, Lee WM. Acetaminophen toxicity in an urban county hospital. *New England Journal of Medicine* 1997; **337**: 1112–1118.

37 Schiødt FV, Lee WM, Bondesen S, Ott P, Christensen E. Influence of acute and chronic alcohol intake on the clinical course and outcome in acetaminophen overdose. *Alimentary Pharmacology & Therapeutics* 2002; **16**: 707–715.

38 Kuffner EK, Dart RC, Bogdan GM, Hill RE, Casper E, Darton L. Effect of Maximal Daily Doses of Acetaminophen on the Liver of Alcoholic Patients: A Randomized, Double-blind, Placebo-Controlled Trial. *Archives of Internal Medicine* 2001; **161**: 2247–2252.

39 Dart RC, Kuffner EK, Rumack BH. Treatment of pain or fever with paracetamol (acetaminophen) in the alcoholic patient: a systematic review. *American journal of therapeutics* 2000; **7**: 123–34.

40 Miners J, Attwood J, Birkett B. Determinants of acetaminophen metabolism: effect of inducers and inhibitors of drug metabolism on acetaminophen’s metabolic pathways. *Clin Pharmacol Ther* 1984; **35**: 480–486.

41 Prescott L, Critchley J, Balali‐Mood M, Pentland B. Effects of microsomal enzyme induction on paracetamol metabolism in man. *British Journal of Clinical Pharmacology* 1981; **12**: 149–153.

42 Perucca E, Richens A. Paracetamol disposition in normal subjects and in patients treated with antiepileptic drugs. *British journal of clinical pharmacology* 1979; **7**: 201–6.

43 Young CR, Mazure CM. Fulminant Hepatic Failure From Acetaminophen in an Anorexic Patient Treated With Carbamazepine. *J Clin Psychiatry* 1998; **59**: 622–622.

44 Parikh S, Dillon LC, Scharf SL. Hepatotoxicity possibly due to paracetamol with carbamazepine. *Internal Medicine Journal* 2004; **34**: 441–442.

45 Jickling G, Heino A, Ahmed SN. Acetaminophen toxicity with concomitant use of carbamazepine. *Epileptic Disorders* 2009; **11**: 329–332.

46 Smith JAE, Hine ID, Beck P, Routledge PA. Paracetamol Toxicity: is Enzyme Induction Important? *Human Toxicology* 1986; **5**: 383–385.

47 *Joint Formulary Committee. British National Formulary.* December 2016 ed. BMJ Group and Pharmaceuticle Press: London, 2016.

48 Kalsi SS, Wood DM, Waring WS, Dargan PI. Does cytochrome P450 liver isoenzyme induction increase the risk of liver toxicity after paracetamol overdose? *Open Access Emerg Med* 2011; **3**: 69–76.

49 Makin AJ, Wendon J, Williams R. A 7-year experience of severe acetaminophen-induced hepatotoxicity (1987–1993). *Gastroenterology* 1995; **109**: 1907–1916.

50 Bock KW, Wiltfang J, Blume R, Ullrich D, Bircher J. Paracetamol as a test drug to determine glucuronide formation in man. Effects of inducers and of smoking. *Eur J Clin Pharmacol* 1987; **31**: 677–683.

51 Kostrubsky SE, Sinclair JF, Strom SC, Wood S, Urda E, Stolz DB *et al.* Phenobarbital and Phenytoin Increased Acetaminophen Hepatotoxicity Due to Inhibition of UDP-Glucuronosyltransferases in Cultured Human Hepatocytes. *Toxicol Sci* 2005; **87**: 146–155.

52 Minton NA, Henry JA, Frankel RJ. Fatal Paracetamol Poisoning in an Epileptic. *Human Toxicology* 1988; **7**: 33–34.

53 Brackett CC, Bloch JD. Phenytoin as a Possible Cause of Acetaminophen Hepatotoxicity: Case Report and Review of the Literature. *Pharmacotherapy: The Journal of Human Pharmacology and Drug Therapy* 2000; **20**: 229–233.

54 Suchin SM, Wolf DC, Lee Y, Ramaswamy G, Sheiner PA, Facciuto M *et al.* Potentiation of Acetaminophen Hepatotoxicity by Phenytoin, Leading to Liver Transplantation. *Dig Dis Sci* 2005; **50**: 1836–1838.

55 Bray GP, Harrison PM, O’Grady JG, Tredger JM, Williams R. Long-Term Anticonvulsant Therapy Worsens Outcome in Paracetamol-Induced Fulminant Hepatic Failure. *Human & Experimental Toxicology* 1992; **11**: 265–270.

56 Rumack BH. Acetaminophen Hepatotoxicity: The First 35 Years. *Journal of Toxicology: Clinical Toxicology* 2002; **40**: 3–20.

57 Niemi M, Backman JT, Fromm MF, Neuvonen PJ, Kivistö KT. Pharmacokinetic Interactions with Rifampicin. *Clin Pharmacokinet* 2003; **42**: 819–850.

58 Stephenson I, Qualie M, Wiselka MJ. Hepatic failure and encephalopathy attributed to an interaction between acetaminophen and rifampicin. *Am J Gastroenterol* 2001; **96**: 1310–1311.

59 Nolan CM, Sandblom RE, Thummel KE, Slattery JT, Nelson SD. Hepatotoxicity Associated With Acetaminophen Usage in Patients Receiving Multiple Drug Therapy for Tuberculosis. *Chest* 1994; **105**: 408–411.

60 Toes MJ, Jones AL, Prescott L. Drug interactions with paracetamol. *American journal of therapeutics* 2005; **12**: 56–66.

61 Crippin JS. Acetaminophen hepatotoxicity: potentiation by isoniazid. *Am J Gastroenterol* 1993; **88**: 590–592.

62 Murphy R, Swartz R, Watkins PB. Severe acetaminophen toxicity in a patient receiving isoniazid. *Annals of internal medicine* 1990; **113**: 799–800.

63 Zand R, Nelson SD, Slattery JT, Thummel KE, Kalhorn TF, Adams SP *et al.* Inhibition and induction of cytochrome P4502E1-catalyzed oxidation by isoniazid in humans. *Clinical Pharmacology & Therapeutics* 1993; **54**: 142–149.

64 Chien JY, Peter RM, Nolan CM, Wartell C, Slattery JT, Nelson SD *et al.* Influence of polymorphic N‐acetyltransferase phenotype on the inhibition and induction of acetaminophen bioactivation with long‐term isoniazid. *Clinical Pharmacology & Therapeutics* 1997; **61**: 24–34.

65 Døssing M, Wilcke JT, Askgaard DS, Nybo B. Liver injury during antituberculosis treatment: an 11-year study. *Tuber Lung Dis* 1996; **77**: 335–340.

66 Larson AM. Acetaminophen Hepatotoxicity. *Clinics in Liver Disease* 2007; **11**: 525–548.

67 Manyike PT, Kharasch ED, Kalhorn TF, Slattery JT. Contribution of CYP2E1 and CYP3A to acetaminophen reactive metabolite formation. *Clinical Pharmacology & Therapeutics* 2000; **67**: 275–282.

68 Diaz D, Fabrev I, Daujat M, Aubert BS, Bories P, Michel H *et al.* Omeprazole is an aryl hydrocarbon-like inducer of human hepatic cytochrome P450. *Gastroenterology* 1990; **99**: 737–747.

69 Farrell G, Murray M. Human cytochrome P450 isoforms. Their genetic heterogeneity and induction by omeprazole. *Gastroenterology* 1990; **99**: 885–889.

70 Xiaodong S, Gatti G, Bartoli A, Cipolla G, Crema F, Perucca E. Omeprazole Does Not Enhance the Metabolism of Phenacetin, a Marker of CYP1A2 Activity, in Healthy Volunteers. *Therapeutic Drug Monitoring* 1994; **16**: 248–250.

71 Sarich T, Kalhorn T, Magee S, Al‐Sayegh F, Adams S, Slattery J *et al.* The effect of omeprazole pretreatment on acetaminophen metabolism in rapid and slow metabolizers of S‐mephenytoin. *Clinical Pharmacology & Therapeutics* 1997; **62**: 21–28.

72 Zevin S, Benowitz NL. Drug Interactions with Tobacco Smoking. *Clin Pharmacokinet* 1999; **36**: 425–438.

73 Bock KW, Schrenk D, Forster A, Griese EU, Mörike K, Brockmeier D *et al.* The influence of environmental and genetic factors on CYP2D6, CYP1A2 and UDP-glucuronosyltransferases in man using sparteine, caffeine, and paracetamol as probes. *Pharmacogenetics* 1994; **4**: 209–218.

74 Prescott LF. Kinetics and metabolism of paracetamol and phenacetin. *Br J Clin Pharmacol* 1980; **10 Suppl 2**: 291S–298S.

75 Schmidt LE, Dalhoff K. The impact of current tobacco use on the outcome of paracetamol poisoning. *Alimentary Pharmacology & Therapeutics* 2003; **18**: 979–985.

76 Miller LG. Recent Developments in the Study of the Effects of Cigarette Smoking on Clinical Pharmacokinetics and Clinical Pharmacodynamics. *Clin-Pharmacokinet* 1989; **17**: 90–108.

77 Itoh H, Nagano T, Hayashi T, Takeyama M. Ranitidine Increases Bioavailability of Acetaminophen by Inhibiting First-Pass Glucuronidation in Man. *Pharmacy and Pharmacology Communications* 2000; **6**: 495–500.

78 Jack D, Thomas M, Skidmore IF. Ranitidine and paracetamol metabolism. *The Lancet* 1985; **326**: 1067.

79 Hazai E, Vereczkey L, Monostory K. Reduction of Toxic Metabolite Formation of Acetaminophen. *Biochemical and Biophysical Research Communications* 2002; **291**: 1089–1094.

80 Mitchell MC, Schenker S, Speeg KV. Selective inhibition of acetaminophen oxidation and toxicity by cimetidine and other histamine H2-receptor antagonists in vivo and in vitro in the rat and in man. *J Clin Invest* 1984; **73**: 383–391.

81 Chen M, Lee C. Cimetidine—Acetaminophen Interaction in Humans. *The Journal of Clinical Pharmacology* 1985; **25**: 227–229.

82 Slattery JT, McRorie TI, Reynolds R, Kalhorn TF, Kharasch ED, Eddy AC. Lack of effect of cimetidine on acetaminophen disposition in humans. *Clinical Pharmacology & Therapeutics* 1989; **46**: 591–597.

83 Abernethy DR, Greenblatt DJ, Divoll M, Ameer B, Shader RI. Differential effect of cimetidine on drug oxidation (antipyrine and diazepam) vs. conjugation (acetaminophen and lorazepam): prevention of acetaminophen toxicity by cimetidine. *J Pharmacol Exp Ther* 1983; **224**: 508–513.

84 Bredfeldt JE, von Huene C. Ranitidine, acetaminophen, and hepatotoxicity. *Ann Intern Med* 1984; **101**: 719.

85 Burkhart KK, Janco N, Kulig KW, Rumack BH. Cimetidine as adjunctive treatment for acetaminophen overdose. *Human & Experimental Toxicology* 1995; **14**: 299–304.

86 Poulsen HE, Ranek L, Jørgensen L. The influence of disulfiram on acetaminophen metabolism in man. *Xenobiotica* 1991; **21**: 243–249.

87 Liu Y, Ramírez J, Ratain MJ. Inhibition of paracetamol glucuronidation by tyrosine kinase inhibitors. *British Journal of Clinical Pharmacology* 2011; **71**: 917–920.

88 Kim D-W, Tan EY, Jin Y, Park S, Hayes M, Demirhan E *et al.* Effects of imatinib mesylate on the pharmacokinetics of paracetamol (acetaminophen) in Korean patients with chronic myelogenous leukaemia. *British Journal of Clinical Pharmacology* 2011; **71**: 199–206.

89 Weise AM, Liu CY, Shields AF. Fatal Liver Failure in a Patient on Acetaminophen Treated with Sunitinib Malate and Levothyroxine. *Annals of Pharmacotherapy* 2009; **43**: 761–766.

90 Burger DM, Meenhorst PL, Koks CHW, Beijnen JH. Pharmacokinetics of Zidovudine and Acetaminophen in a Patient on Chronic Acetaminophen Therapy. *Annals of Pharmacotherapy* 1994; **28**: 327–330.

91 Shriner K, Goetz MB. Severe hepatotoxicity in a patient receiving both acetaminophen and zidovudine. *The American Journal of Medicine* 1992; **93**: 94–96.

92 Mitchell JR, Thorgeirsson SS, Potter WZ, Jollow DJ, Keiser H. Acetaminophen-induced hepatic injury: Protective role of glutathione in man and rationale for therapy. *Clinical Pharmacology & Therapeutics* 1974; **16**: 676–684.

93 Pirotte J. Apparent potentiation of hepatotoxicity from small doses of acetaminophen by phenobarbital. *Ann Intern Med* 1984; **101**: 403–403.
